# ‘They don’t know what to do with our children’: Experiences and views on feeding and swallowing from parents of children who use long-term ventilation

**DOI:** 10.1177/13674935241242824

**Published:** 2024-04-09

**Authors:** Sabrena Lee, Jeanne Marshall, Michael Clarke, Christina Smith

**Affiliations:** 1 Evelina London Children’s Hospital, Guy’s and St Thomas’ Hospital NHS Foundation Trust, 325312 University College London, London, UK; 2 325312 Faculty of Brain Sciences, University College London, London, UK; 3 Queensland Children’s Hospital, 1974 Children’s Health Queensland Hospital and Health Service, Queensland, Australia; 4 School of Health and Rehabilitation Sciences, The University of Queensland, Queensland, Australia; 5 7147 San Francisco State University, San Francisco, CA, USA; 6 Department of Clinical Neurosciences, NHS Lothian, Edinburgh, Scotland

**Keywords:** Paediatrics, respiration, artificial, deglutition disorders, feeding methods, qualitative research, parents

## Abstract

Increasing use of paediatric long-term ventilation (LTV) has been reported around the world over the last two decades and it is anticipated that use of this medical intervention will continue to grow. Research has shown that children who use LTV have risk factors for feeding and swallowing difficulties which result in long-term reliance on non-oral feeding methods. This Patient and Public Involvement (PPI) activity explored experiences of parents of children with LTV on their children’s feeding and swallowing journeys. Individual and group interviews with seven parents were conducted. Interview data was then analysed using content analysis. Families discussed a range of themes including impacts on their family, facilitators and barriers to feeding and swallowing journeys, speech and language therapy (SLT) support, their family’s healthcare journey in relation to quality of life and future directions for research. This study highlighted potential key areas to explore when identifying ways to improve SLT care and research in feeding and swallowing for children who use LTV.

## Introduction

Long-term ventilation (LTV) is defined as dependence on respiratory support for longer than 3 months and once an individual is medically stable (National Confidential Enquiry into Patient Outcome and Death, 2020). Parents of children using LTV juggle being a caregiver, advocate, activist, educator and case manager (Carnevale et al., 2006). Many aspects of their lives are complicated and often overwhelming (Carnevale et al., 2006). Despite this, there is limited information available about families’ experiences and views on their children’s feeding and swallowing journeys.

Children who use LTV present with complex medical needs that encompass a range of sleep and respiratory challenges (Brookes, 2021; Codesal et al., 2015). The prevalence of feeding difficulties in this population is not known; however, it has been estimated that 50% of children with medical conditions present with feeding and/or swallowing issues (Larson-Nath and Goday, 2019). Children with LTV often have oral secretion management issues, oral aversion, gagging, prolonged chewing, difficulty transitioning to harder textures, swallowing difficulties and risk of aspiration (Abraham and Wolf, 2000; Bagnall et al., 2006; Brooks et al., 2021; Chen et al., 2021; Fuchimoto et al., 2011; Kawaguchi et al., 2005; Roper and Quinlivan, 2010; Shah and Jadcherla, 2020). The presentation of these difficulties commonly results in long-term reliance on non-oral feeding methods (Goneidy et al., 2022).

The complex health issues that children face require constant and demanding care from caregivers and the multidisciplinary team (Edwards et al., 2020). Children with LTV and their families perceive their quality of life (QOL) to be lower than the general population and children with other chronic illnesses (Mattson et al., 2022). Children reported feeling restricted by their technological equipment, whereas families expressed anxiety and high levels of responsibility for their children’s health (Mattson et al., 2022). However, the impact of children’s feeding difficulties on their families’ lives has not been thoroughly explored in research literature. Therefore, we lack insight into the day-to-day challenges that parents face in this area. Whilst a range of stressors (such as their children’s health needs, social isolation, and challenges with normality and stability at home) can contribute to parental distress (Carnevale et al., 2006), this paper will focus only on the impact of feeding and swallowing on patients and their families.

Research with children without LTV has shown that feeding and nutrition difficulties increase levels of caregiver stress (Silverman et al., 2021). Children’s developmental delays, mealtime behaviour challenges, aggressive behaviours and mood/anxiety contributed to parental stress (Silverman et al., 2021). Parents of intensive care survivors reported that feeding became the central focus of their lives and resulted in family tension and social isolation (Morton et al., 2019). Parents who provide medical care at home found nutrition-related procedures emotionally and technically challenging (Page et al., 2020). Non-oral feeding has significant psychosocial impacts on children and families (Remijn et al., 2022). Gastrostomy feeding regimens are demanding and time-consuming, and impact on parents’ social activities (Brotherton et al., 2007).

As medical technology improves and survival rates of children with complex health needs increases, use of LTV continues to rise (National Confidential Enquiry into Patient Outcome and Death, 2020). It is important for health professionals to understand impacts of LTV on children’s feeding journeys. National service planning in this area has emphasised the importance of increasing access to multidisciplinary care and reducing variability in therapy services across settings (National Confidential Enquiry into Patient Outcome and Death, 2020). It is anticipated that families have unique and compelling views and experiences about these topics. They are therefore central to shaping planning for future clinical and research needs, and their contributions are vital in identifying key research priorities and facilitating the improvement of service provision across the field of paediatric LTV (NHS England, 2014).

Patient and Public Involvement (PPI) is defined as research that is carried out with or by the public rather than to or for them (National Institute of Health and Care Research, 2021). PPI can improve the quality and applicability of research as it facilitates active partnership between researchers and relevant patient groups (National Institute of Health and Care Research, 2021). As there is little research available about feeding and swallowing in children with LTV, PPI is an ideal method to explore the current issues and challenges that patients and families face. In turn, outcomes from PPI activity can inspire future research planning, and therefore improve service delivery and patient care.

## Aims

To inform research priorities and needs in the topic of feeding and swallowing.

## Method

### Design

PPI activity was conducted between January 2022 to September 2022. PPI research is distinct from typical qualitative work as it conducts research with patients and the public, instead of using them as subjects (National Institute of Health and Care Research, 2021). PPI facilitates active partnership between patients, stakeholders and researchers (National Institute of Health and Care Research, 2021).

Semi-structured interviews and focus groups were conducted. These methods were chosen to encourage open discussion, obtain a broad range of views and experiences, and allow for sensitive topics to be explored. This work was conducted in collaboration with WellChild charity, a national UK charity for children with long-term and complex medical needs and their families. WellChild charity has longstanding experience in conducting research activities and has a large network of families with LTV needs. Two WellChild representatives, a charitable programmes project manager and a family engagement manager, were involved during design, recruitment and interview stages. They disseminated advertisements for recruitment, recruited participants, distributed participant information sheets, collected consent forms and convened group interviews.

### Ethics and data protection

Ethical approval for this project was obtained from University College London and registered with the data protection office (registration number: Z6364106/2022/02/46 health research). Participants received a £10 shopping voucher.

### Recruitment

Any parent or carer of children with LTV needs was eligible to participate. Inclusion criteria included parents or carers with children aged 18 years and younger residing in the UK. Exclusion criteria included those who did not provide consent to participate. Sample size estimation was approximately 10 to 15 participants. Convenience sampling was conducted and advertisement of study recruitment occurred through social media (e.g., Twitter and Facebook) and email across paediatric health care teams. Participant registration was completed on WellChild’s website. Applicants were provided with a choice of two dates for focus group interviews. Those who were unable to attend on those dates were offered an individual interview. Eligible participants received an information sheet and consent form outlining research procedures, including recording of interviews and anonymity of data.

### Procedure

An interview topic guide was written by the research team (see supplementary material). Interviews were conducted on Zoom (Zoom Video Communications, Inc. (version 5.14.10)). Focus groups were led by SL and convened by WellChild representatives. WellChild representatives introduced SL but did not contribute to interview discussion. Interviews ran for 30 to 75 min. Confidential information was removed from transcriptions. Participants were assigned a pseudonym to maintain their anonymity.

### Data analysis

Interviews were analysed using inductive content analysis. Content analysis provides descriptive reporting of surface meaning of interviews and is beneficial for exploring emerging research topics (Graneheim and Lundman, 2004). This method was therefore most suitable for addressing the objectives of this PPI activity. The team met with three researchers, working within the same university departments as the research team and experienced in qualitative data analysis, to ensure that content analysis was the most appropriate method.

Content analysis classifies qualitative information into words, categories and themes to determine key meanings and patterns (Vaismoradi et al., 2013). It involves preparation, organisation and reporting of information (Graneheim and Lundman, 2004). Analysis focused on manifest content (words directly observed in interviews rather than the underlying interpretation) (Graneheim and Lundman, 2004). Interview transcripts were combined into one unit of analysis. Then, two team members separately organised one focus group transcript into condensed meaning units and codes. Two team members met to discuss findings, confirm coding patterns, and agree on underlying categories and themes. This set of patterns was applied to the remaining unit of analysis, with a plan to confer with the team in the case of additional codes emerging. Final analysis findings were discussed between all team members.

### Rigour and trustworthiness

Planning, implementation and reporting of research activity followed the ‘Consolidated Criteria for Reporting Qualitative Research (COREQ)’ checklist (Tong et al., 2007) (see supplementary material). Following data analysis, a summary of findings and an anonymous online survey were sent to participants to check their agreement of identified themes, categories and codes. This feedback allowed for additional credibility of research findings and facilitated ongoing engagement with the participants as a valued PPI group.

## Findings

### Interview process

**Figure 1.**
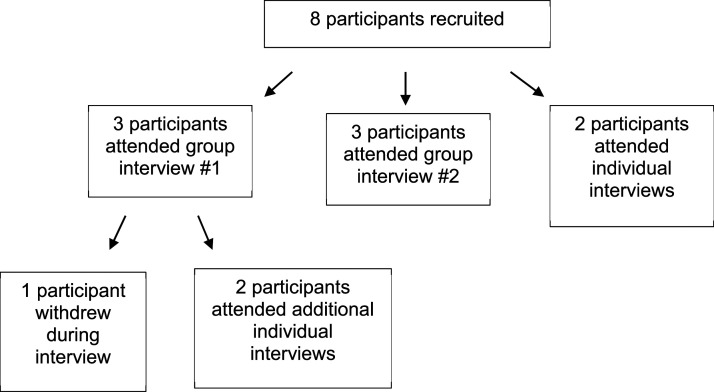
Summary of the interview process. The interview process included two group interviews and four individual interviews in total.

Eight participants were recruited. One participant withdrew at the beginning of the focus group as they felt they could not contribute to the research topic. Information from this participant was not included in data analysis. Initially, two focus group interviews, followed by two individual interviews, took place. All participants were informed that they could request an additional individual interview if they would like to provide further information about the topics discussed in their initial interview. Two focus group participants contacted WellChild representatives to request additional individual interviews. These participants wanted to provide more information about the research topic. Therefore, a total of two group interviews and four individual interviews were conducted (see Figure 1). All interviews were completed in March 2022.

### Participant demographics

All seven participants were mothers of children with LTV needs. One parent had two children with LTV needs, therefore eight children were discussed in the interviews. See Table 1.

**Table 1.** Table of participant demographics.

Demographic	Categories	Number of parents (*N* = 7) n (%)
Sex	Male	0 (0%)
	Female	7 (100%)
Ethnicity	White British	7 (100%)
Age	35–39 years	2 (29%)
	40–44 years	2 (29%)
	45–49 years	3 (43%)
Highest level of education	Diploma	1 (14%)
	Bachelor’s degree	4 (57%)
	Master’s degree	2 (29%)

*Note.* The table above provides demographic information, including sex, ethnicity, age and highest level of education, of participants.

### Medical and LTV characteristics

Participants’ children had a range of medical backgrounds including genetic, cardiac, prematurity, respiratory, neuromuscular and structural airway conditions. These medical needs are representative of the wider population of children with LTV but did not encompass all potential respiratory and neurological conditions that children with LTV may experience. LTV needs are summarised in Table 2.

**Table 2.** Summary of children’s demographics, LTV, and feeding and swallowing characteristics.

Demographic, LTV and feeding characteristics	Categories	Number of children (*N* = 8) n (%)
Sex	Male	6 (75%)
	Female	2 (25%)
Age	0–3 years	1 (12.5%)
	3 years 1 month–6 years	3 (37.5%)
	6 years 1 month–9 years	2 (25%)
	9 years 1 month–12 years	1 (12.5%)
	12 years 1 month–15 years	1 (12.5%)
Type of ventilation	Invasive	6 (75%)
	Non-invasive	2 (25%)
Duration of ventilation in 24-h period	Nocturnal	4 (50%)
	24 h	3 (37.5%)
	Not stated	1 (12.5%)
Previous use of non-oral feeding method	No	2 (25%)
	Yes	6 (75%)
Current use of non-oral feeding method	No	3 (37.5%)
	Yes	5 (62.5%)
Current feeding regime	Blended diet via gastrostomy	3 (37.5%)
	Oral diet with supplemental gastrostomy feeds	1 (12.5%)
	Formula feeds via nasogastric tube and oral tastes of purees	1 (12.5%)
	Full oral diet	3 (37.5%)
Current feeding and swallowing difficulties	Oral secretion management issues	1 (12.5%)
	Oral motor difficulties	3 (37.5%)
	Oral aversion	2 (25%)
	Dysphagia	1 (12.5%)
	Food refusal due to sensory sensitivities	2 (25%)
	Food neophobia	2 (25%)
	Risk of aspiration on fluids/food	1 (12.5%)
	None reported	1 (12.5%)

*Note.* Some children presented with multiple feeding and swallowing difficulties; sensory sensitivities included wet food, food of particular shapes, food of particular textures.

### Feeding and swallowing characteristics

Children’s feeding and swallowing characteristics are provided in Table 2. Reasons for non-oral feeding were unsafe swallow, medical instability, gastrointestinal issues and hydration. Oral motor difficulties, including difficulty with tongue movement, chewing and use of open cups were frequently reported. Oral aversion and food refusal due to sensory sensitivities were also common. Occasional coughing and choking on food or drink were mentioned by two parents although they were not concerned about risk of aspiration. Two children experienced difficulties with bottle feeding and breastfeeding due to reduced feeding endurance and increased respiratory effort.

### Content analysis: Themes

Five primary themes were derived from the data: (1) impacts on family, (2) facilitators and barriers, (3) SLT support, (4) family’s healthcare journey in relation to QOL and (5) future directions. See Table 3 for themes, categories and codes.

**Table 3.** Themes, categories and codes identified across interviews.

Theme	Category	Codes
Impact of child’s feeding and swallowing needs on family	Family dynamics and bonding	• Siblings unable to feed child
		• Siblings do not understand feeding difficulties
		• Family unable to treat child in the same way as siblings
		• Parent unable to bond with child through feeding
		• Family unable to use food as ‘treats’
	Social participation	• Difficulty eating out at restaurants
		• Difficulty engaging with other children
		• Judgement from strangers
		• Extra time taken to prepare meals
		• Limited knowledge from the public
	Emotional stressors	• Guilt about what child cannot do
		• Expressing breastmilk when child was not feeding orally
		• Emotional impact of feeding and swallowing difficulties on child
		• Emotions regarding child’s feeding and swallowing journey
		• Difficult seeing other parents feed their children
		• Finding information was emotionally draining
	Additional time and resources	• Training in non-oral feeding methods
		• Implementation of feeding and swallowing strategies
		• Incorporating non-oral feeding into daily routine
		• Received support from health professionals outside of SLT profession
Facilitators and barriers to child’s feeding and swallowing journey	Facilitators	• Providing nutrition through blended diet
		• Secretion management intervention
		• Parent’s professional background
		• Learning about food in a variety of settings
		• Early intervention
		• Support from family members
		• Managing feeding regime
		• Information from other families
		• Accessed and used information available online
		• Non-oral feeding method took pressure off oral feeding
	Barriers	• Prioritisation of breathing over feeding
		• Limited opportunities for pre-feeding and oral feeding practice
		• Limited guidance and parental education
		• SLT sessions only targeted communication skills
		• Hospital environment
		• SLT expertise is lacking regarding LTV
		• Information available online is confusing and limited
		• Limited information from other health professionals
SLT support for feeding and swallowing	Assessment and intervention	• Reason for instrumental assessment
		• Instrumental assessment results
		• Type of intervention
		• Parent completed intervention without SLT support
	Service provision	• Parent felt supported by SLT input
		• Parent did not feel supported by SLT input
		• Timing of intervention
		• No SLT on LTV team
		• SLT on LTV team
		• Frequency of SLT services
		• Barriers to accessing services
Family’s healthcare journey in relation to LTV	Misalignment of child’s progress and health professionals’ expectations	• Child is doing better than expected medically
		• Health professionals told parent that child had limited potential for improvement
		• Parent felt need to push for services
	Quality of life	• LTV has improved quality of life
		• Non-oral feeding has improved quality of life
		• Difficulty communicating about feeding and swallowing needs and wants
		• Child/family have been accepting of feeding and swallowing journey
		• Negative impact of feeding and LTV equipment on quality of life
Future directions	Suggestions for future LTV and feeding/swallowing care	• SLT in every LTV team
		• Being more open-minded to oral feeding potential
		• Early intervention
		• Addressing emotional stressors from feeding and swallowing journey
		• Improved access to information on LTV is needed
		• Research in this area

*Note.* This table lists the themes, categories and codes identified during data analysis of all interviews.

Five parents responded to the post-interview online survey and all parents agreed with identified themes (see supplementary material).

### Impacts of child’s feeding and swallowing needs on family

Family dynamics and bonding was discussed by all participants. Negative impacts of children’s feeding difficulties on parents, siblings and grandparents were raised. Parents missed out on important feeding experiences including bonding, using food as treats and involving siblings in feeding routines. Families experienced difficulty eating at restaurants as children were unable to eat common items on restaurant menus due to delayed feeding development and/or narrow food repertoires.

“It's little things for us, I think, as a family. Like, we love going out to eat and we have to think very carefully about where we go.” (Colleen)

“Because if you go anywhere, he can’t just eat something off a menu. You can’t just order some chips or some pasta because he wasn’t eating those things maybe by that point, so it's just always having to think ‘oh, well, where can we buy the cheesy pie from?’ or he started eating a Greek yogurt but he’ll only have a particular type one or only a particular brand of hummus.” (Fiona)

Parents reported limitations in social participation for oral and non-oral feeders.

“Socially it has a massive impact. They can’t, you know, both boys sit at the table at school and nursery with the kids but they're not engaging.” (Elizabeth)

“He doesn’t eat often around lots of other children because of everything that's happened.” (Fiona)

Elizabeth described the additional preparation involved in making and packing her children’s blended diet meals for gastrostomy feeds:

“Restaurants and places of interests or attractions aren’t geared for tube-fed children.” (Elizabeth)

Parents described feelings of grief, sadness and guilt related to their children’s loss of skills, feeding difficulties and need for non-oral feeding.

“Hearing that he potentially wouldn’t be able to eat anything at all orally absolutely broke me.” (Georgia)

“You kind of grieve those different aspects that you're losing as time goes on, and it is a grieving process really, for us, because these things are being taken away.” (Anne)

The additional time and resources required for implementing strategies (with and without SLT support) was reported.

“You know how difficult and challenging it is day to day, three meals a day and snacks, it's like constant, like, the grind is hard.” (Fiona)

### Facilitators and barriers

#### Facilitators

The three most frequently encountered codes included (a) early intervention, (b) information from other families and (c) accessed and used information available online. Families whose children received SLT support felt that early intervention was a key contributor to their children’s progress. Early intervention resulted in positive impacts for children’s chewing skills, introduction to oral feeding and expansion of food repertoire.

“You know, after I'd got that food play lady, that was all I needed really. I was away then. I could follow the little pattern. I had her number in case I had any questions and it really was so easy. I had to clean the kitchen floor about three times a day, but you know, just like I would never have thought that driving trucks through Coco Pops whilst not trying to eat the stuff was going to make such a vast difference, but that was what it was in the end that made (child) totally eat and it's a really easy thing.” (Diana)

Fiona described her gratitude for early intervention in the hospital setting during her child’s prolonged and complex stay as a baby.

“I think we'd have missed opportunities if I was just starting with tasting things when we got home. He wouldn't be eating now, definitely not, and then those challenges I’ve explained to you as a mother would have been greater and we'd still be having huge difficulties now, whereas we're pumping the air because he's doing really well.” (Fiona)

Families commonly sought information about intervention through websites and online family forums. Colleen “googled a lot” as her child never received feeding support from a SLT. Fiona also sourced online resources as she felt she needed more intervention ideas than those provided by the hospital and community SLTs.

#### Barriers

The most frequently encountered barriers were (a) lack of SLT expertise regarding LTV and (b) limited information from other health professionals. Parents commonly felt that SLTs did not demonstrate confidence in their knowledge of LTV-related feeding and swallowing management.

“I think we struggle with the uniqueness of them [children with LTV needs] because people often don’t know what to do and as a result, they often don’t know how to help.” (Elizabeth)

“It's not their [SLTs] fault because they're not exposed to these children, but it just felt like they hid, rather than came to see us.” (Colleen)

“And you find that when we do see them [SLTs], they’ll pick one of the others to focus on, so they'll focus on his speech and the feeding's just ‘well, we'll sit him at the table and at some point, he'll just eat’. And I think there's just got to be something more to speech and language therapy than putting them at the table and expecting them to eat.” (Elizabeth)

Parents discussed the difference in services across hospital and community teams. They felt there was more LTV expertise in the hospital setting. Parents often relied on the hospital SLT for specialist-related advice, even when their child was supported by the community team.

“We were still in touch with (hospital) as well because obviously I was very trusting in them. They knew (child), they knew the long-term ventilated side of trying to eat and having the power to do all these sorts of things that I was trusting in them because they had all the information and had supported us for a long time.” (Fiona)

“We end up split between the two, so we go to (hospital) SLT for some things and have (community) SLT for some things, which doesn’t really help the situation.” (Elizabeth)

Frustrations in sourcing advice from other health professionals within LTV and/or specialist medical teams were described. Parents wished other health professionals had identified potential areas for improvement in oral feeding.

“If they would have said, ‘you know what, actually, I bet, you know, if he’s tolerating a bag of crisps, maybe we could look at some of the strategies to help you’ and if there would have been that proactiveness once you knew it was safe, then that would have been really brill’.” (Diana)

“They don’t know what to do with our children is my overwhelming feeling. Then again, like (child), he’s very unique, he doesn’t really fit in the box, and I just get, you know, ‘we don’t know what to do with you’.” (Colleen)

### SLT support

#### Assessment and intervention

Five children had an instrumental swallowing assessment in the form of a videofluoroscopic swallow study (VFSS). Reasons for VFSS included recurrent chest infections and assessment of swallowing safety on fluid textures. Three parents reported that their children showed adequate swallowing. Despite this, two children’s oral feeding plans were not progressed following the VFSS. Elizabeth reported that her older child showed safe swallowing but displayed ‘tracheostomy-related’ issues, and her younger child was still awaiting readiness for a VFSS. Georgia reported that her child showed signs of unsafe swallowing on liquid and pureed textures.

“Just before we left (hospital), they did all of the swallow tests and they said that he could swallow everything but at that time, to be honest, he was really unstable and I think the focus was on the breathing bit. So, he did swallow everything in the test, but they didn't want to, at that point, try to do it.” (Diana)

“We’re in a slightly maddening position with our younger boy (child) whereby when he goes to nursery, all of the children have like a sippy cup for their drink and (child) wants one but we’re not allowed to let him drink from it ‘cause he’s not had a swallow assessment and you can’t have a swallow assessment until he’s having three spoonfuls of proper food. So he’ll pretend to do it with an empty cup that he’s given. And this has been the case for months, whereby I’ve got a child who wants to have a drink but he’s not allowed to, which means we’re going around in circles.” (Elizabeth)

SLT support included parental education, introduction to oral tastes, chewing strategies and systematic desensitisation feeding therapy. Feeding therapy using systematic desensitisation involves gradual exposure to a feared stimulus (i.e., food and drink) using activities intended to promote relaxation (e.g., play) (Marshall et al., 2013). Only one parent reported that thickened fluids were used briefly when her child was in hospital. Parents never received support for oral cares or oral secretion management from an SLT.

Parents commonly completed intervention without SLT support. These parents used baby-led weaning strategies and sensory-based feeding therapy sourced from the internet and family forums.

“Although we didn't have any formal play therapy, we very much went down the case of ‘look we're just going to, when we eat, we're going to put something there and if you throw it on the floor that's fine and if you play with it that's fine’. And I think it took us probably about 18 months for him to get to the point where some of it went in his mouth.” (Colleen)

#### Service provision

Four parents felt supported by the SLT during their child’s hospital admission. Six parents wished for more support from their local SLT service in the community setting.

“I can’t figure out for the life of me what priority is over and above a child who can’t speak and who can’t eat but clearly there is because we don’t seem to get the therapy.” (Elizabeth)

Reasons that parents did not feel supported included limited contact from the SLT team, limited support for intervention, confusion about the role of the SLT and lack of LTV knowledge.

“(Child) will lick food. I don’t know if then he, I don’t know, takes a bite of a cucumber, what I’m meant to do in that situation. Do I let him swallow it? Do I fish it out of his mouth and give him an even greater oral aversion? It’s really hard to know what to do best to be honest.” (Elizabeth)

Timing of SLT intervention varied greatly, however always commenced after initiation of LTV (see Table 4). One child never received SLT support and two children were on the waiting list for community SLT feeding services.

**Table 4.** Summary of timing of SLT intervention.

Onset of feeding and swallowing difficulties	Age at initiation of SLT support	Setting of support	Current SLT support
7 years	7 years	School	Yes
At birth	6 months	Hospital	No
At birth	No support received	No support received	No
At birth	3 years	Community	No
At birth	Not stated	Hospital	No
At birth	Not stated	Hospital	No
At birth	3 months	Hospital	Yes
At birth	8 years	Community	No

*Note.* Abbreviations used in the table include SLT (speech and language therapy).

Frequency of SLT services received was variable. Parents voiced their frustrations about their limited access to support. Only three children’s multidisciplinary LTV teams included an SLT.

“We've been on and off their list. We are currently off their list, but we've been on and off it, and I keep referring myself back in.” (Diana)

“It’s horrifically isolating that there is nowhere I would go and pick up the phone … because we don’t know who to turn to.” (Colleen)

One parent removed her child’s nasogastric tube at home without any SLT input. She recalled:

“I said to my community nurse, I said ‘I can’t wait for speech and language anymore, they just put us on the backburner or something. I can’t get hold of anyone.’” (Barbara)

Other barriers included Coronavirus disease 2019 pandemic, long waiting lists, geographical location, and limited attendance at school due to illness, and care package funding.

### Family’s healthcare journey in relation to LTV

#### Misalignment of child’s progress and health professional’s expectations

Children’s oral feeding and/or medical progress had exceeded their healthcare team’s expectations.

“She’s a very happy little girl and she’s proved a lot of people wrong with her tracheostomy and ventilation.” (Anne)

Health professionals told parents that their children had limited potential to develop oral feeding skills.

“I'm frustrated the whole way through (child)’s early development because whether it was speech and language or walking or anything ‘cause I was always told like ‘Oh, you know, don't really think he's going to make it’ and I just thought you know at least give us the tools and let us have a go.” (Diana)

Families therefore felt the need to advocate for health services in hospital and community settings.

“Normally, parents are passive in that environment where they're not sure what's happening. The medical staff are the ones giving them directions on what's happening, so you don't really challenge, and we saw that a lot and I think if I didn't push, (child) probably wouldn't be where he is now.” (Fiona)

### QOL

LTV generally improved family’s families QOL. Families described mixed feelings regarding non-oral feeding. Two parents stated that introducing a blended diet via gastrostomy improved their child’s QOL. Another parent felt the gastrostomy made mealtimes more positive for the family. One child disliked and pulled out his feeding equipment. One parent adapted LTV equipment for bike riding. Children had a range of communication difficulties, which at times impacted on their ability to voice their feelings about their feeding needs.

“There was never any sort of behavioural, you know, crying or anything like that really, definitely not around the eating. It just became just became part of, you know, the journey.” (Anne)

“She’s grew up with it [LTV], she thinks it's normal, so it's not something that that wasn't there and then was there… we just don’t know no different with her because she’s had it for so long.” (Barbara)

### Future directions

Families expressed the importance of having access to SLT support within their LTV team. Parents whose children had not received support wished they were provided with early intervention and felt this would have been key to improving their oral feeding skills.

“I look back now and it’s totally obvious that we would have ended up where we are, i.e., with two children with very significant oral aversion because we were never given that opportunity when they were younger to really focus on, not necessarily eating if we weren’t able to at that stage, but focus on food play. Priority was always respiratory side and I think well of course we’re going to end up here because they were never given that opportunity.” (Elizabeth)

Parents also wished that SLTs recognised their children’s potential for improvement in oral feeding.

“There’s almost an assumption locally that children like ours won’t eat. That seems to be the starting point and I just think they need to re-think that. If they just come to it with a slightly more open mind, I think a lot more could be done.” (Diana)

All parents were thankful to the research team for their interest in this topic and agreed that further research was needed in this area.

“The reason I requested this interview with you is because I’ve never even heard of LTV and swallowing as a pull-together research. No one’s ever done it. Why’s no one ever done it? They tend to come hand-in-hand, don’t they?” (Georgia)

## Discussion

This PPI activity provided novel insight into the views of parents of children with LTV and oral feeding needs. The activity aim was achieved through engagement with a PPI group who openly discussed experiences that shaped their children’s feeding and swallowing journeys. Five main themes emerged and highlighted issues that need addressing in future research planning.

Children presented with oral feeding characteristics similar to those described within the literature. However, some children had outcomes that exceeded expectations described in current research (Goneidy et al., 2022). Half of the children transitioned to full oral feeding or oral feeding with supplemental gastrostomy feeds during their childhood. Early intervention and improving access to resources were felt to be most impactful to children’s outcomes.

Children’s feeding difficulties had impacts across personal, family, social and health-related facets of their family’s lives. Studies exploring the experiences of parents of children with LTV report similar findings. Even parents of children without LTV experience higher levels of stress and increased risk of anxiety and depression due to their children’s feeding difficulties (Fernández De Valderrama Rodríguez et al., 2022). Negative perceptions from family, friends and the public are also common for families of children with non-oral feeding needs (Brotherton et al., 2007; Fernández De Valderrama Rodríguez et al., 2022; Simione et al., 2020).

The emotional toll of children’s feeding difficulties resulted in grief, sadness and guilt for parents. They strongly felt that SLT support should include addressing parents’ emotions. An increased need for psychological support is common for families of children with disabilities and complex health problems (Whiting, 2013). It has previously been recognised that parents of children with chronic feeding disorders would benefit from screening and support for stress (Silverman et al., 2021).

SLT service delivery was inconsistent in timing, frequency and type of intervention. Similarly, parents of paediatric and neonatal intensive care unit survivors found that discharge education about promoting and maintaining feeding skills was lacking (Morton et al., 2019). Limited expertise in paediatric LTV was encountered most, especially for those without access to a tertiary specialist LTV SLT. This is consistent with recent research that reported that a minority of LTV services contained SLTs (National Confidential Enquiry into Patient Outcome and Death, 2020). SLTs should be equipped with necessary skills and resources to provide timely and optimal support.

VFSS is considered gold standard assessment for objective swallowing assessment (Lo Re et al., 2019). Parents felt confused about management plans following VFSS. Good communication between SLTs and parents is important to ensure understanding of assessment results and recommendations. Modifying fluid and food textures is commonly used to improve feeding skills or safety (Dodrill and Gosa, 2015). Only one parent recalled thickened fluids and/or modified foods being trialled. There is currently no standard assessment or intervention protocol for this population despite the complex factors that LTV pose on oral feeding.

Parents reported varying levels of perceived SLT confidence and skill. This highlights the need to examine current education and training goals across clinical settings. Despite receiving limited support, parents were determined to provide oral feeding therapy using online resources and information from other families with LTV.

### Limitations

PPI activity allowed researchers to obtain first-hand views and experiences of children and families’ feeding and swallowing journeys. Completing both individual and group interviews allowed for rich and robust discussion. However, the PPI cohort comprised of a small self-selected sample of mothers in one country. Mothers and fathers of children with a disability show differences in their roles, expression of emotions and relationships with health professionals (Pelchat et al., 2003). Future PPI work should involve participants from a variety of ages, ethnic groups, gender and social backgrounds to ensure diversity and accurate representation of public experience and insight (NIHR Centre for Engagement and Dissemination, 2021).

The convenience and low cost of online interviews may have increased recruitment. However, the use of technology was not inclusive of all social groups. Building rapport and obtaining emotional information for research purposes can be challenging when conducted over videoconference (Khan and MacEachen, 2022). Likewise, those who attended individual interviews may have felt more comfortable divulging in-depth and sensitive information than those in the focus groups.

### Implications for practice

This research activity provides an opportunity to reflect on the current practice of assessment of feeding and swallowing in children with LTV. Services should prioritise early intervention by advocating for early referral to SLT teams. Timely use of VFSS can lead to a reduction in the risk of chest infections (Lo Re et al., 2019). It is therefore an important diagnostic tool for this population. Parents were motivated to improve their children’s oral feeding skills despite the challenges they faced. They should be empowered to be central to children’s intervention plans (Reeder and Morris, 2021). Providing appropriate resources, support and education could unlock opportunities for oral feeding success. Evaluating current training needs within the SLT profession is vital to ensure that clinicians are equipped with the skills and knowledge required to deliver optimal services.

### Conclusions

The experiences and views of parents of children with LTV are essential to informing and enhancing services. A range of topics were discussed in detail in interviews and focus groups, all highlighting the need for improvement in SLT services across several key areas. This work is an initial step in facilitating ongoing clinical academic research regarding the feeding outcomes of children with LTV. Future research objectives should target understanding the patient’s feeding and swallowing journey in relation to SLT support needs, service use and experiences. This will allow researchers to investigate the facilitators and barriers from the family’s and health professional’s perspectives. This would be foundational in contributing to the planning of SLT care for children with LTV and thereby improve the overall healthcare journey for these children in years to come.

## Supplemental Material

Supplemental Material - ‘They don’t know what to do with our children’: Experiences and views on feeding and swallowing from parents of children who use long-term ventilation Supplemental Material for ‘They don’t know what to do with our children’: Experiences and views on feeding and swallowing from parents of children who use long-term ventilation by Sabrena Lee, Jeanne Marshall, Michael Clarke and Christina Smith in Journal of Child Health Care
