# Supplementary material for: ‘They don’t know what to do with our children’: Experiences and views on feeding and swallowing from parents of children who use long-term ventilation
Source: J Child Health Care. 2024 Apr 9;29(4):842–60. doi: 10.1177/13674935241242824 (PMC12662838; doi:10.1177/13674935241242824)
Supplement: Supplemental Material - ‘They don’t know what to do with our children’: Experiences and views on feeding and swallowing from parents of children who use long-term ventilation [file sj-pdf-1-chc-10.1177_13674935241242824.pdf]

Supplementary Material File 1:
Completion of Consolidated Criteria for Reporting Qualitative Studies (COREQ) Checklist

**Domain and Personal Characteristics**	**Achieved? (Description)**
Domain 1: Research Team and Reflexivity<br>• Personal Characteristics<br>• Relationship with Participants	SL was involved in all stages of the project, including the design, implementation, interpretation, and writing of the manuscript.<br>• Occupation: Speech and Language Therapist<br>• Gender: Female<br>• Training: Bachelor of Speech Pathology, Masters of Research<br>• Relationship established prior to research: No<br>• Participant knowledge of researcher: Researcher information (including name, workplace and research goals) listed on recruitment advertisement<br>• Interviewer characteristics: Reasons and interest in research topic<br><br>JM was involved in the design of the project, interpretation of the data, and in preparation of the manuscript via draft reviews.<br>• Occupation: Speech Pathologist<br>• Gender: Female<br>• Training: Bachelor of Speech Pathology, PhD<br>• Relationship established prior to research: No<br>• Participant knowledge of researcher: Supervisory<br>• Interviewer characteristics: N/A (not involved in interviews)<br><br>MC was involved in the design of the project and in preparation of the manuscript via draft reviews.<br>• Occupation: Speech and Language Therapist and Assistant Professor<br>• Gender: Male<br>• Training: Speech and Language Therapy MSc, Speech Sciences PhD<br>• Relationship established prior to research: No<br>• Participant knowledge of researcher: Supervisory<br>• Interviewer characteristics: N/A (not involved in interviews)<br><br>CS was involved in the design of the project, interpretation of the data, and in preparation of the manuscript via draft reviews.

	• Occupation: Associate Professor / Speech and Language Therapist<br>• Gender: Female<br>• Training: Bsc, MSc, Ph.D.<br>• Relationship established prior to research: No<br>• Participant knowledge of researcher: Supervisory<br>• Interviewer characteristics: N/A (not involved in interviews)
Domain 2: Study Design<br>• Theoretical Framework<br>• Participant Selection<br>• Setting<br>• Data Collection	• Theoretical framework: content analysis<br>• Selection of participants: Convenience sampling<br>• Participants approached using online website, social media and email<br>• Number of participants: 7<br>• Participants who withdrew: 1 participant as they felt they could not answer the research question<br>• Data collected: Home (online via web conferencing platform 'Zoom') with audio and visual recording conducted and field notes taken during interviews<br>• Presence of non-participants: WellChild representatives<br>• Interview guide provided in Appendix A<br>• Repeat interviews conducted: No<br>• Data saturation discussed: Yes<br>• Transcripts returned to participants: No
Domain 3: Analysis and Findings<br>• Data Analysis<br>• Reporting	• Number of data coders: 3<br>• Description of coding tree provided: Yes<br>• Derivation of themes provided: Yes<br>• Software used: Microsoft Excel and Microsoft Office<br>• Participant checking: Yes, one-page summary sent to all participants and post-study survey completed by participants<br>• Quotations provided: Yes and identified<br>• Data and findings consistent: Yes<br>• Presentation of major themes: Yes<br>• Clarity of minor themes: Yes

Supplementary Material File 2:

PPI Activity Topic Guide

1) Firstly, let's go around and introduce ourselves by name. Can you please say how old your child is and very briefly if you they have had any feeding or swallowing difficulties before?
   - *Prompts:*
     - *Is your child currently eating or drinking by mouth? Do they need any thickened fluids or modified diets?*
     - *Does your child have a feeding tube?*
     - *How has your child's eating and drinking difficulties impacted on your family's life?*

2) Please put your hand up if your child has seen a speech and language therapist (SLT) before? Would anyone like to comment on what their experience has been like?
   - *Prompts:*
     - *Has your child's eating or drinking difficulties gotten better over time? What helped with this?*
     - *If your child has been referred to a SLT before, was the referral process easy/hard?*
     - *How long did you have to wait to see a SLT after the referral?*
     - *Has your child seen a SLT in the hospital, school, or community setting?*
     - *If your child hasn't seen a SLT before, have any professionals recommended SLT support for eating/drinking support for your child?*

3) When experiencing speech and language therapy support in the hospital or community or school setting, have there been any aspects that you feel is lacking or needs improving?
   - *Prompts:*
     - *Do you feel you understand the role of the SLT in feeding and swallowing? Has this been clearly explained to you by your LTV team?*
     - *Do you have multiple SLTs?*
     - *How has communication between professionals been across settings?*
     - *Do you prefer the SLT support in one setting over another?*
     - *Does your SLT work closely with the specialist respiratory/LTV team in your tertiary hospital?*
     - *What aspects of your child's SLT support are you happy with / unhappy with?*
     - *What are the barriers to accessing SLT support? E.g. frequent hospital admissions / lack of service in your local area / long waiting times*

4) As a parent or carer, what is meaningful to you to know more about in the areas of feeding and swallowing and LTV?
- *Prompts:*
  - *What would be useful for other parents to know when facing these challenges?*
  - *In what ways do you think we can improve experiences for others so they don't have to experience the same challenges?*
  - *What has been the most stressful part of the eating/drinking journey for your child and your family?*
  - *What information do you wish your LTV team told you more about when it comes to feeding and swallowing?*
  - *When you have questions about feeding and swallowing, where do you find answers? E.g. Do you ask your SLT? Do you look up information on the internet? Is your LTV team helpful?*

5) When thinking about ideas for future research, are there any topics or ideas that come to mind?
   - *Prompts:*
     - *For example, there are many ways we can improve services. This can be through creating resources for families or health professionals, improving our current feeding/swallowing assessments and interventions, or completing surveys from parents about the current issues in this area, just to name a few.*
     - *If we were to create resource packages about feeding/swallowing for families, which information do you think is important for these packages to contain?*
     - *If we were to create resource packages for health professionals, which information do you think is important for these packages to contain?*
     - *If we were to improve SLT feeding/swallowing assessment and intervention, what do you think we could improve? Do you think current assessment is accurate and reflective of your child's skills? Do you think SLT intervention is helpful for your child's skills?*

- *If we were to conduct surveys, what do you feel is important for health professionals to know about how parents/carers feel?*

Supplementary Material File 3:

Parent Feedback Survey and Results

Theme and Category	Current experience n (%)	Previous experience n (%)	Not part of family's experience n (%)	Additional comments
Impact of Child's Feeding and Swallowing Needs on Family (Social Participation)	3 (60%)	2 (40%)	0 (0%)	
Impact of Child's Feeding and Swallowing Needs on Family (Family Dynamics and Bonding)	2 (40%)	2 (40%)	1 (20%)	• "No siblings"
Impact of Child's Feeding and Swallowing Needs on Family (Emotional Stressors)	3 (60%)	1 (20%)	1 (20%)	• "I can see how this would concern other parents but it was not a concern of mine"
Impact of Child's Feeding and Swallowing Needs on Family (Additional Time and Resources)	3 (60%)	2 (40%)	0 (0%)	• "Also extra washing and time needed to physically eat is much longer than peers"
Facilitators and Barriers to Child's Feeding and Swallowing Journey (Facilitators)	3 (60%)	2 (40%)	0 (0%)	
Facilitators and Barriers to Child's Feeding and Swallowing Journey (Barriers)	3 (60%)	2 (40%)	0 (0%)	
SLT Support for Feeding and Swallowing (Assessment and Intervention)	3 (60%)	2 (40%)	0 (0%)	
SLT Support for Feeding and Swallowing (Service Provision)	4 (80%)	1 (20%)	0 (0%)	• "After 14 years on LTV, we have only just in the last 9 months been taken on as a patient under an LTV team"

				• "My child did not receive feeding and swallowing intervention at school, despite this being written in their Education, Health and Care Plan"
Family's Healthcare Journey in Relation to LTV	4 (80%)	1 (20%)	0 (0%)	• "Also very much around family and access to things other take for granted i.e. meals out"
Future Directions	3 (60%)	2 (40%)	0 (0%)	

Note. The table above provides the results of the online parent survey and feedback questionnaire. All answers were anonymous.
